# Switchgrass PvDREB1C plays opposite roles in plant cold and salt tolerance in transgenic tobacco

**DOI:** 10.1186/s41065-017-0050-4

**Published:** 2017-10-24

**Authors:** Wuwu Wen, Zheni Xie, Guohui Yu, Chengliang Zhao, Jing Zhang, Linkai Huang, Bin Xu, Bingru Huang

**Affiliations:** 1 College of Agro-grassland Science, Nanjing Agricultural University, Nanjing, 210095 People’s Republic of China; 2 Department of Grassland Science, Animal Science and Technology College, Sichuan Agricultural University, Ya’an, Sichuan 625014 People’s Republic of China; 3 Department of Plant Biology and Pathology, Rutgers, the State University of New Jersey, New Brunswick, NJ 08901 USA

**Keywords:** Switchgrass, DREB/CBF, Dual function, Cold, Salt, Stress

## Abstract

**Background:**

The C-repeat-binding factors/DRE-binding factors (CBF/DREBs) comprise a key transcription factor family involved in plant stress tolerance. Yet, there is limited information about switchgrass DREB genes and their functional roles.

**Results:**

In this study, four cold-inducible *PvDREB1s* were identified from switchgrass (*Panicum virgatum*), among which *PvDREB1C* was the one responded to cold stress later than the other three *PvDREB1s*. Yet, ectopic overexpression of *PvDREB1C* led to significantly compromised, instead of improved cold tolerance in transgenic tobacco. On the other hand, *PvDREB1C* was transcriptionally down-regulated in response to salt stress, but overexpression of *PvDREB1C* improved plant salt tolerance in transgenic tobacco. The improved salt tolerance was associated with increased K^+^/Na^+^ ratio and Ca^2+^ content, higher cellular osmotic potential, and activation of stress-related functional genes in the leaves of transgenic plants under salt stress.

**Conclusions:**

The current results implied that *PvDREB1C* played opposite roles in plant cold and salt tolerance. Although DREB1s were known as positive stress regulators, particular attentions shall be paid to their potential negative regulatory role(s).

**Electronic supplementary material:**

The online version of this article (10.1186/s41065-017-0050-4) contains supplementary material, which is available to authorized users.

## Background

Switchgrass (*Panicum virgatum*) is a perennial, C_4_ tall grass used for biofuel and forage feedstock production, water and soil conservation, as well as habitat restoration [1–3]. Switchgrass is known for its wide adaptation to diverse and relatively harsh environment [4]. However, genetic improvement of switchgrass is highly desirable to successfully establish switchgrass in marginal lands with minimal biomass sacrifice and little cost of resource and labor inputs.

The C-repeat-binding factors/DRE-binding factors (CBF/DREBs) constitute an indispensable regulatory network for plant tolerance against abiotic stresses (e.g. cold, drought and salinity, etc.) [5]. DREBs primarily function through transactivating the expression of downstream functional genes through binding to the C-repeat/dehydration responsive (CRT/DRE) cis-elements [6]. Previous studies revealed that CBFs play important roles in plant cold tolerance through the IEC1-CBF regulon to activate the expression of downstream cold-responsive genes [7]. CBF/DREBs (e.g. OsDREB1D and OsDREB1F) were also reported to positively regulate plant salt and drought tolerances [8, 9]. Despite the importance of DREB family genes in plant stress tolerance, there is limited information about switchgrass DREB genes and their functional roles.

The objective of this study was to analyze the biological function of a *DREB1* gene for its potential use in switchgrass genetic improvement. The cloned switchgrass *DREB1C* was up-regulated by cold stress but down-regulated by salt and osmotic stress. Yet, over-expression of *PvDREB1C* in transgenic tobacco led to compromised cold tolerance, but improved salt tolerance with lower Na^+^/K^+^ ratio and higher reactive oxygen scavenging enzyme activities under salt stress.

## Results

### Four switchgrass *DREB1* genes transcriptionally responsive to cold exposure

**Fig. 1:**
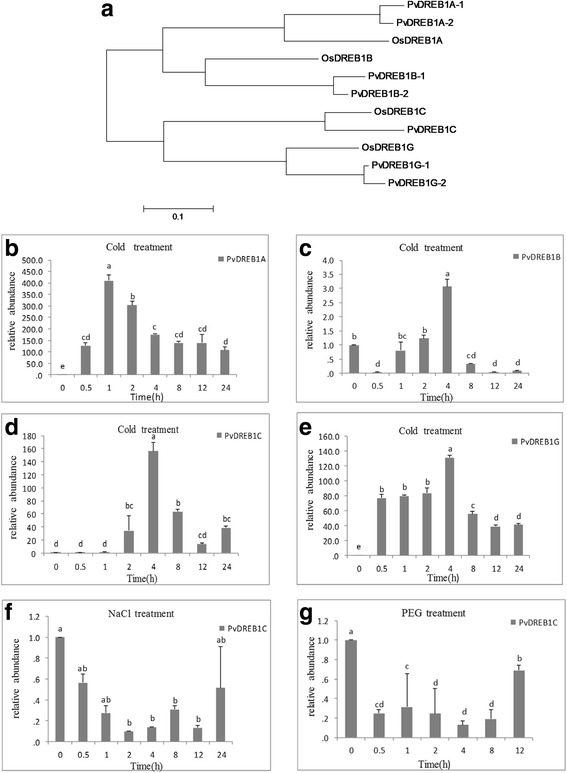
Phylogenetic analysis and relative expression of four cold-inducible PvDREB1s. **a** The Neighbor-Joining tree was built using MEGA6 software. The optimal tree is drawn to scale, with the sum of branch length = 1.99584622 is shown. **b**-**e** Expression of *DREB1*s in response to low temperature (4 °C). **f**-**g** *PvDREB1C* was transcriptionally suppressed by 250 mM NaCl or PEG-6000 (20%, *W*/*V*). Data are represented as mean ± SE (*n* = 3), and different letters above bars indicate a significant difference at *P* < 0.05

In searching for CBFs/DREB1s involved in cold tolerance in switchgrass, four rice cold-responsive DREB1s [i.e. OsDREB1A (Os09g0522200), OsDREB1B (Os09g0522000), OsDREB1C (Os06g0127100), OsDREB1G (Os02g0677300) [10, 11] were used to search corresponding orthologs against the switchgrass genome database (*Panicum virgatum* v1.1, DOE-JGI) using BLAST. Seven orthologous switchgrass *PvDREB1*s were found according to their corresponding orthologous genes in rice. According to the phylogenetic analysis (Fig. 1a), these *PvDREB1*s were named as *PvDREB1A-1*/*−2* (Pavir.J03891.1 & Pavir.Ba01280.1), *PvDREB1B-1*/*−2* (Pavir.J19655.1 & Pavir.Ba01283.1), *PvDREB1C* (Pavir.J35991.1), and *PvDREB1G-1*/*−2* (Pavir.Ab02685.1 & Pavir.Aa00912.1). Switchgrass ‘Alamo’ is an allotetraploid with two closely related chromosomal sets [12, 13]. *PvDREB1G-1* and −*2* were paralogous genes in corresponding homeologous chromosomes 1b and 1a, respectively (switchgrass genome from JGI: *Panicum virgatum* v4.1). Although the chromosome locations of *PvDREB1A-1*and *PvDREB1B-2* were not determined yet, these two paired *PvDREB1*s were likely derived from the allotetraploidy event of switchgrass as well [12, 13].

To verify whether these *PvDREB1s* were cold-inducible genes, we exposed non-acclimatized switchgrass to 4 °C. The three paired genes shared highly similar nucleotide sequence identity that the paired genes could not be discriminated from each other using real-time PCR. Therefore, the detected transcript levels of *PvDREB1A, PvDREB1B*, or *PvDREB1G* should be the combination of their respective paralogous genes (e.g. the detected transcript level of *PvDREB1A* should be comprised of both *PvDREB1A-1* and *PvDREB1A-2* as shown in Fig. 1). As predicated, these four *PvDREB1*s all responded to low temperature treatment, yet their expression patterns differed. *PvDREB1A* and *PvDREB1G* were quickly induced by the cold treatment that their transcripts dramatically increased over 100 times within 0.5 h and reached its peak level after 1 or 4 h respectively. The expression level of *PvDREB1B* quickly decreased after 0.5 h of cold treatment, then slowly increased and reached its peak after 4 h.

*PvDREB1C* was the one responded relatively late to cold treatment, and its transcripts accumulated after 2 h- of treatment, reached its peak after 4 h and decreased thereafter. In particular, *PvDREB1C* was also transcriptionally suppressed by NaCl and drought treatments within 0.5 h and decreased to its lowest level 2–4 h after the corresponding stress treatment (Fig. 1f, g). Since most *DREB1*s genes were transcriptionally up-regulated in response to abiotic stresses [5], it was uncommon to see that the transcription of *PvDREB1C* was decreased under salt and drought stresses. Therefore, the function of PvDREB1C was further investigated.

### Ectopic overexpression of *PvDREB1C* in tobacco negatively affected its tolerance against cold stress

**Fig. 2:**
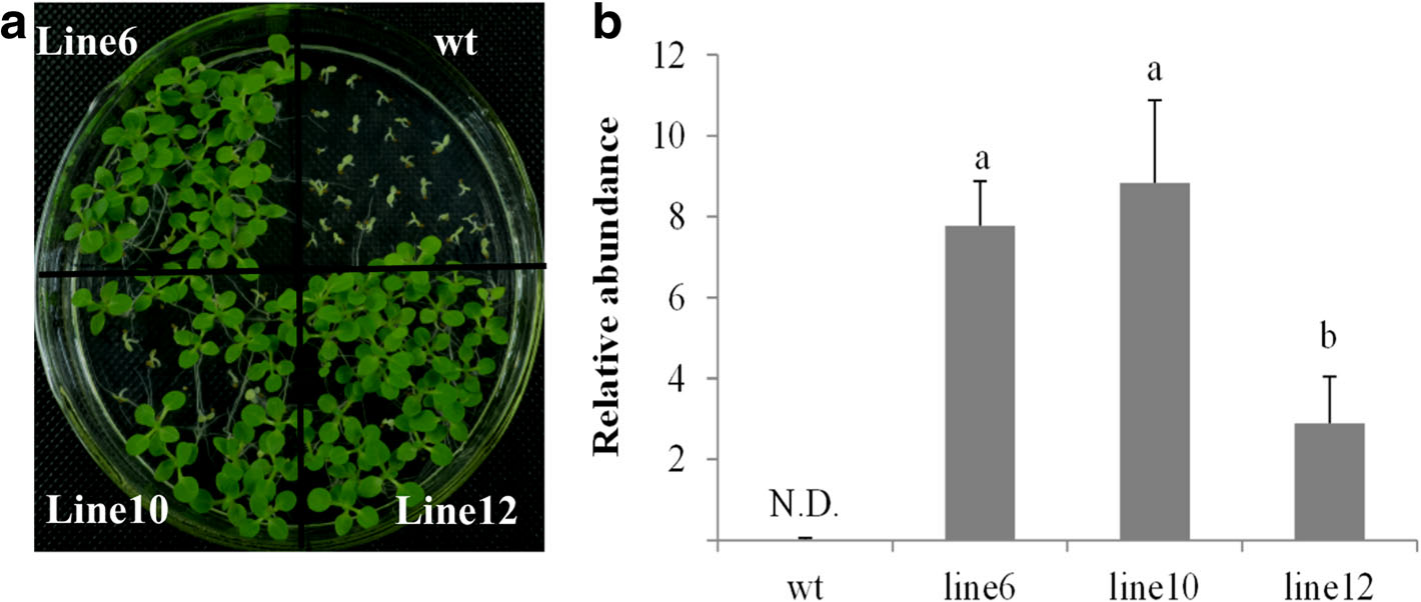
Verification of transgenic lines used in this study. **a** T2 seeds germinated in selective medium containing 15 mg∙L^−1^ bialaphos. **b** Relative expression of the transgene, *PvDREB1C*, in three transgenic lines. Data are represented as mean ± SE, and different letters above bars indicate a significant difference at *P* < 0.05

**Fig. 3:**
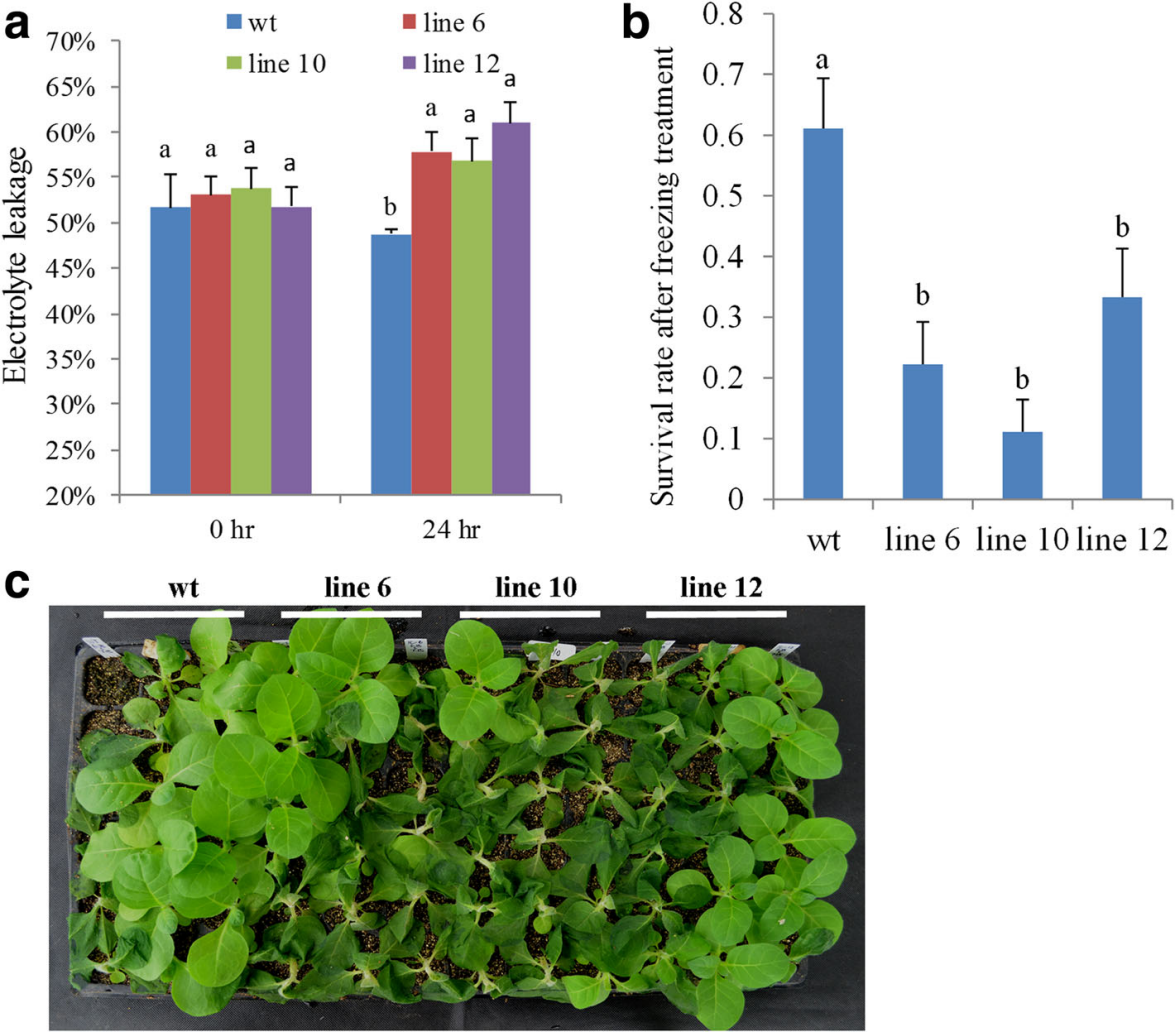
Overexpressing *PvDREB1C* compromised the cold tolerance in tobacco. **a** Electrolyte leakage rates of tobacco exposed to 4 °C for 24 h. **b**, **c** Survival rate and phenotype of tobacco 2 days after −4 °C treatment for 3 h. Data are means of 18 plants of each line with two independent experimental repeats. Data are represented as mean ± SE, and different letters above bars indicate a significant difference at P < 0.05

Stable transgenic tobacco plants were generated to further characterize the function of *PvDREB1C*. As shown in Fig. 2, three transgenic lines with varied expression levels of *PvDREB1C* were used for analysis. These transgenic plants did not show apparent phenotypic alterations compared to wildtype (WT) plants under the optimum condition. However, the transgenic plants showed higher electrolyte leakage (EL) rates under chilling stress (4 °C) (Fig. 3a), and had significantly lower survival rates than those of WT when exposed to freezing stress (−4 °C) (Fig. 3b-c), demonstrating that over-expression of *PvDREB1C* led to compromised cold tolerance.

### *PvDREB1C* improved plant salt tolerance in tobacco

**Fig. 4:**
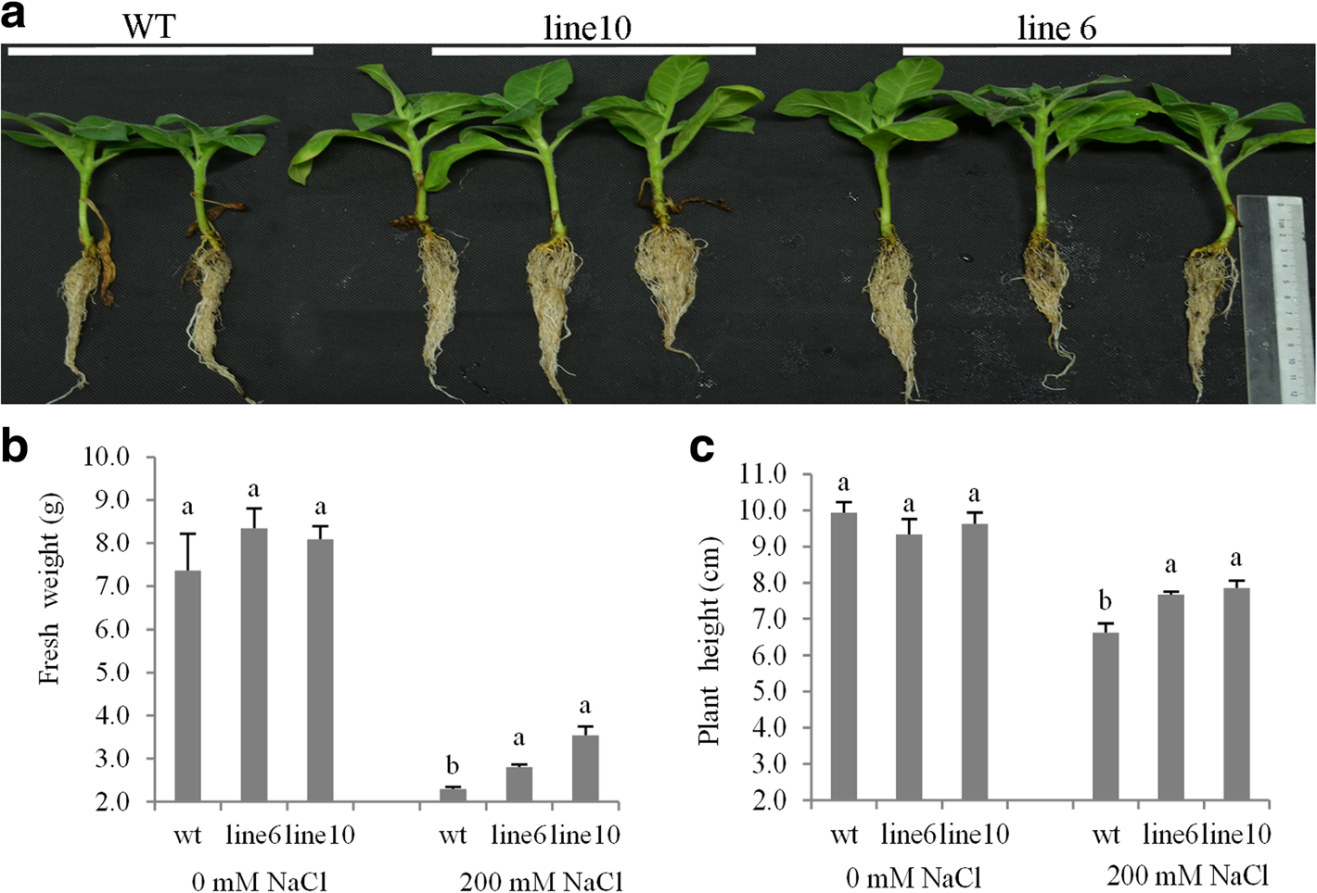
Transgenic *PvDREB1C* tobacco showed improved tolerance to salt stress. **a-c** The *PvDREB1C* transgenic plants showed better performance after 25 days of 200 mM NaCl treatment: phenotypes **a**, fresh weight **b** and plant height **c** of transgenic and wildtype plants. Data are represented as mean ± SE (*n* = 6), and different letters above bars indicate a significant difference at *P* < 0.05

Since the expression of *PvDREB1C* was inhibited by PEG and NaCl treatments, we further tested whether transgenic plants had altered drought and salt tolerance. Unchanged drought tolerance was observed with the transgenic plants (data not shown), while significantly improved salt tolerance was recorded. As shown in Fig. 4, the transgenic plants had significantly bigger plant sizes with higher fresh weight (FW) and plant height than those of the WT after 25 days of salt treatment by watering with 200 mM NaCl.

**Fig. 5:**
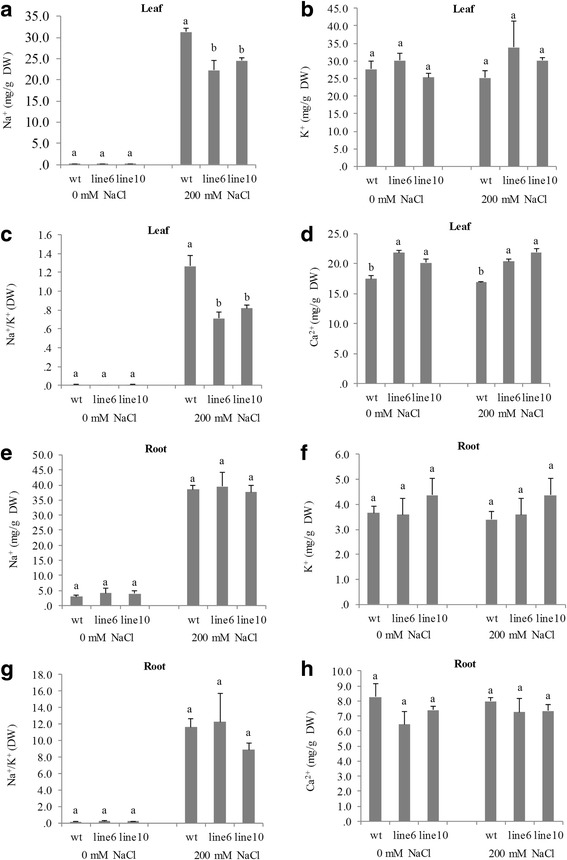
Contents of Na^+^, K^+^ and Ca^2+^, and ratios between Na^+^ and K^+^in leaves **a**-**d** or in roots **e**-**h**. Data are represented as mean ± SE (*n* = 6), and different letters above bars indicate a significant difference at *P* < 0.05

Plants respond to salinity stress through two distinct phases: ion-specific and osmotic-changing phases [14]. Both phases were checked at physiological and gene expression levels comparing the transgenic with the WT plants. Without salt treatment, the transgenic plants had comparable contents of Na^+^ and K^+^ in both leaves and roots with the WT plants; under salt stress, the transgenic plants demonstrated significantly less Na^+^ contents and Na^+^/K^+^, and higher Ca^2+^ contents in leaves but not in roots (Fig. 5), suggesting that overexpressing *PvDREB1C* altered the sodium transportation from root to shoot.

**Fig. 6:**
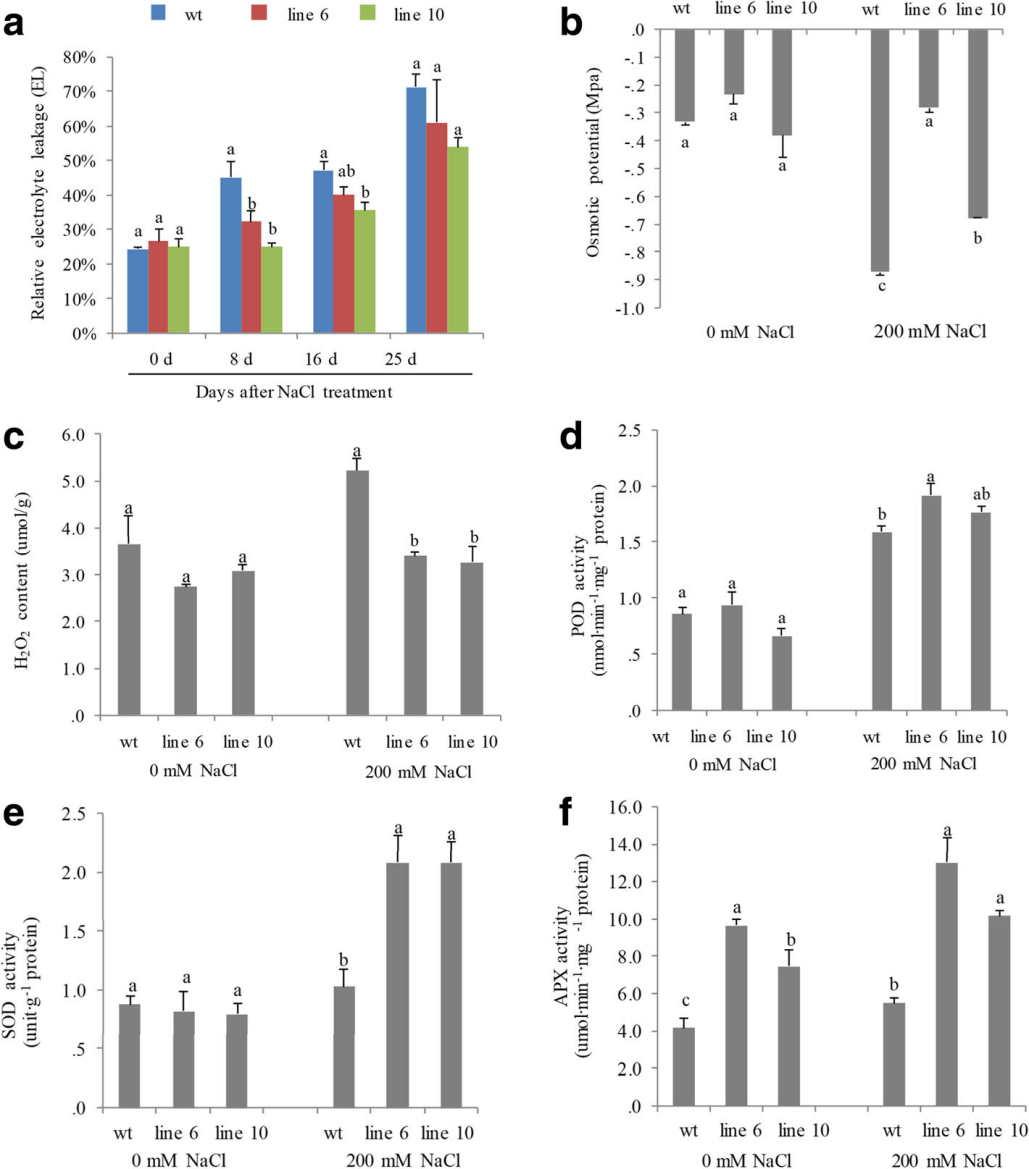
Physiological responses of *PvDREB1C* transgenic and wildtype plants under salt stress. **a** Relative electrolyte leakage (EL) after the first watering with NaCl. **b**-**f** Osmotic potential, H_2_O_2_ content, POD, SOD and APX activities in wildtype and transgenic plants 25 days after NaCl treatment. Data are represented as mean ± SE (n = 6), and different letters above bars indicate a significant difference at *P* < 0.05

At the osmotic-changing phase, the transgenic plants also demonstrated significantly higher osmotic potential, and higher activities of ROS-scavenging enzymes [ascorbate peroxidase (APX), superoxide dismutase (SOD) and peroxidase (POD)], lower H_2_O_2_ contents and smaller EL values (reflecting better cell membrane integrity) than those in the WT under salt stress (Fig. 6).

### Overexpression of *PvDREB1C* activated stress-related functional genes

**Fig. 7:**
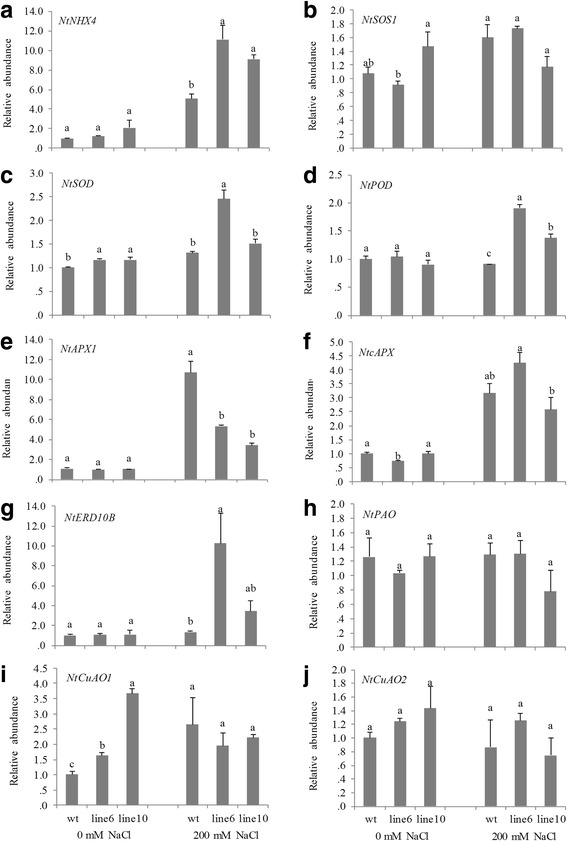
Transcriptional changes of downstream functional genes encoding for ion transporters **a**, **b**, ROS-scavenging enzymes **c**-**f**, dehydrin and polyamines **g**-**j**. Data are represented as mean ± SE (n = 3), and different letters above bars indicate a significant difference at P < 0.05

Relative expression levels of ten downstream functional genes encoding for Na^+^/K^+^ transporters, ROS-scavenging enzymes, dehydrins and polyamines were further checked (Fig. 7). And the qRT-PCR results showed that relative expression levels of one Na^+^/K^+^ transporter gene (*NtNHX4*), two ROS-scavenging enzyme genes (*NtSOD* &*NtPOD*), and a dehydrin-encoding gene (*NtERD10B*) were significantly higher in the transgenic plant than those in the WT under salt stress, while those of *NtSOS1*, *NtcAPX*, *NtPAO*, and *NtCuAO2* were largely unchanged.

**Fig. 8:**
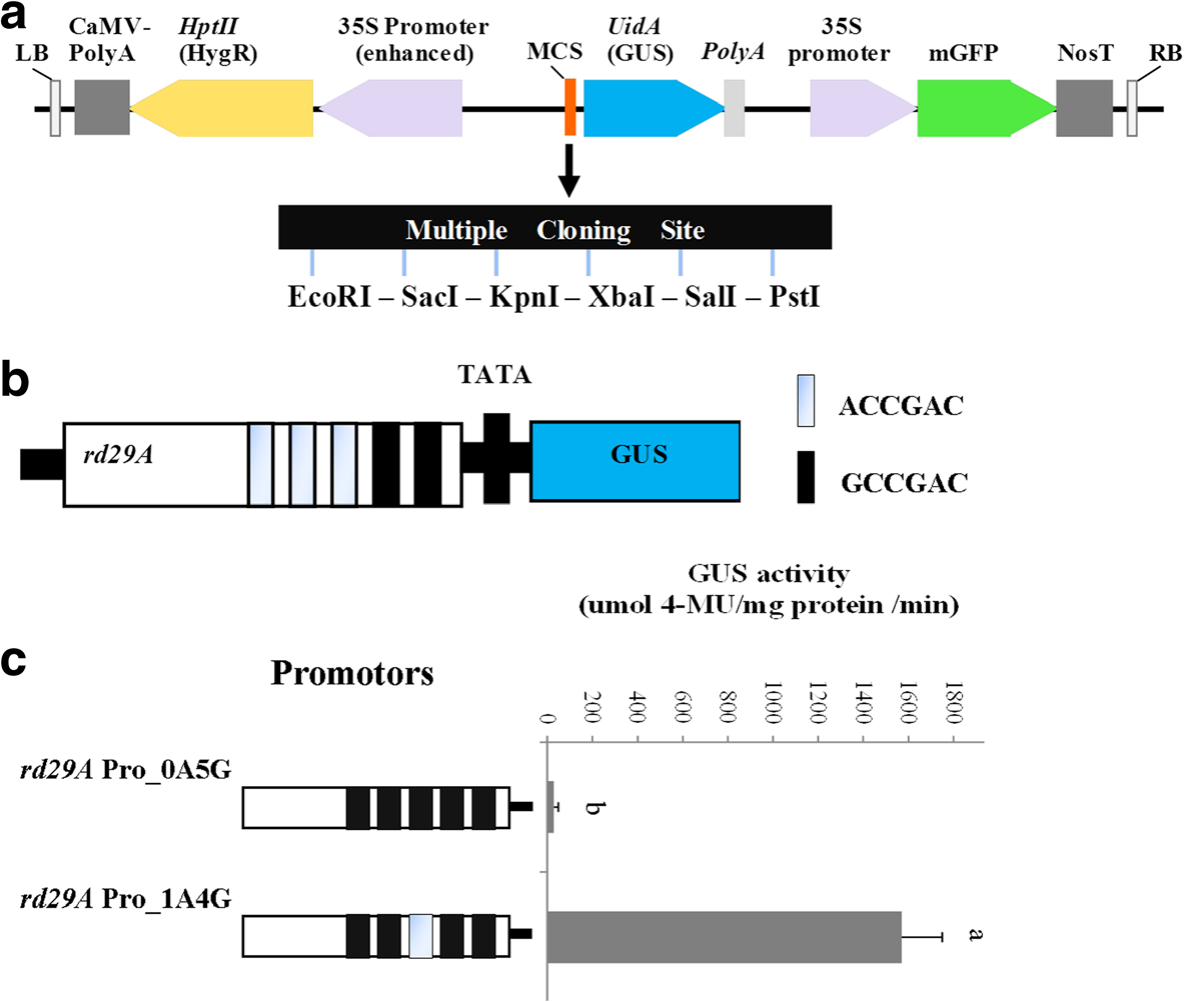
PvDREB1C activated *rd29A* promoter. **a** The pCAMBIA1302-Pro: GUS-polyA vector constructed for promoter analysis. **b** Activation of *rd20A*:*GUS* reporter gene by PvDREB1C as measured with the activity of GUS. Data are represented as mean ± SE (*n* = 15), and different letters above bars indicate a significant difference at *P* < 0.05

It is known that DREB1s preferentially bind to DRE cis-element (ACCGAC or GCCGAC) to activate their downstream genes. The presence of at least one ACCGAC DRE cis-element in -2Kb promoter regions of *NtSOD*, *NtPOD*, and *NtERD10B* were identified (see Additional file 1), suggesting that these genes were among the direct target genes of PvDREB1C. To further understand the binding preference of PvDREB1C to the two types of DRE cis-elements, we generated mutant *rd29A* promoters with one ACCGAC and four GCCGAC (rd29A_pro-1A4G_), or with five GCCGAC but no ACCGAC (rd29A_pro-0A5G_) as illustrated in Fig. 8 (sequences presented in Additional file 1). Co-transformation of 35S_pro_:*PvDREB1C* (effector) and *rd29A*_pro_:*UidA* (reporter) in *N. benthamiana* showed that PvDREB1C activated the expression of *GUS* only in the presence of ACCGAC in the mutant *rd29A* promoter (Fig. 8), suggesting that PvDREB1C preferentially activated those promoters with ACCGAC as the core DRE cis-element.

## Discussion

The CBF/DREB1 transcription factors constitute important signaling components in plant tolerance to various abiotic stresses [15]. In most cases, positive regulatory roles of CBF/DREB1 in plant abiotic stresses were documented [15], and their biological functions were often positively associated with their transcriptional responses to abiotic stresses, such as *DREB1A*/*CBF3* & *DREB1B*/*CBF1* in Arabidopsis [6, 16], and *OsDREB1A* [11] and *OsDREB1B* [17] in rice. One exception was *CBF2*/*DREB1C* in Arabidopsis, which suppressed *CBF1* & *CBF3* and thereby negatively affected plant cold tolerance [18]. Yet, overexpressing the Arabidopsis *DREB1C* improved plant drought tolerance [19], suggesting that CBF2/DREB1C played opposite roles in orchestrating plant tolerance against cold and drought stresses. In this study, we initially identified *PvDREB1C* as a cold-inducible gene, but found that overexpressing *PvDREB1C* lead to compromised plant cold tolerance, unchanged drought tolerance and improved salt-tolerance, suggesting that both *PvDREB1C* and Arabidopsis *DREB1C* had opposite functions in regulating plant tolerance against different types of abiotic stress. Although DREB1s were known as positive stress regulators, particular attentions shall be paid to their potential negative regulatory role(s).

Plants employ different strategies to cope with cold and salt stresses. Yet, crosstalk between these abiotic stress responses via common signaling molecules (e.g. ABA and Ca^2+^) or pathways was recorded [20]. Chilling (above 0 °C) primarily causes the damage of cell membrane system, inhibition of photosynthesis as well as oxidative stress, while freezing (below 0 °C) exerts additional mechanical damage due to the formation of ice crystals within cells or in intercellular spaces [21]. At the molecular level, cold is sensed by membrane proteins such as COLD1, leading to cytosolic Ca^2+^ signal to activate MAP kinase cascade, that phosphorylate transcription factors (e.g. ICE1) [22, 23], and the activated ICE1 rapidly induces the expression of CBF/DREB1s to trans-activate the expression of downstream cold-responsive genes [7]. Ca^2+^ signal also play important roles in plant salt stress tolerance: Na^+^ stress triggers cytosolic Ca^2+^-signal to activate SOS3 (in roots) or SCaBP8/CBL10 (in shoots) that interact with and activate a kinase, SOS2. The activated SOS2 phosphorylates and activates SOS1, a Na^+^/H ^+^ antiporter at the plasma membrane to pump Na^+^ out of cells, thus restoring ion homeostasis [20]. Another set of Na^+^/H^+^ antiporters (NHXs) locate in vacuole membrane to facilitate Na^+^ compartmentalization and maintain intracellular K^+^ status [20]. CBF/DREB1s were also reported to be involved in plant salt tolerance as well. For example, overexpressing *OsDREB1D* and *OsDREB1F* improved plant salt tolerance in rice and/or Arabidopsis possibly by targeting genes involved in ionic and osmotic regulations [8, 9].

The *PvDREB1C* transgenic plants had significantly higher Ca^2+^ contents in leaves but not in roots, and Ca^2+^ activated the Na^+^/H ^+^ antiporters to exclude Na^+^ from leaf cells, thereby reduced the ionic damage caused by Na^+^ on the transgenic plants. At the transcription level, overexpressing *PvDREB1C* activated the expression of *NtNHX4* which was involved in Na^+^ compartmentalization [20], but not the expression of *NtSOS1* which was responsible for pumping Na^+^ out of the cell [20]. The low Na^+^/K^+^ ratio in leaves of transgenic plants was probably due to altered expression of *SOS* genes in below-ground organs rather than in leaves which needs further investigation in the future. On the other hand, the *PvDREB1C* plants also had higher osmotic potential and less H_2_O_2_ accumulation, suggesting that PvDREB1C also functioned in activating osmotic-regulation and ROS-scavenging systems under salt stress [14]. Yet, when subjected to chilling stress, the transgenic lines had higher EL suggesting that their plasma membranes were more vulnerable to cold. The exact reason behind that difference needs to be further investigated in the future (e.g. by understanding the full picture of its downstream target gene networks).

Different DREBs have preference to the specific sequences of the core DRE cis-element. For examples, the rice CBF3/DREB1A bound to GCCGAC more preferentially than to ACCGAC, whereas the Arabidopsis CBF3/DREB1A could efficiently bind to both GCCGAC and ACCGAC [11]. Flanking sequences of the DRE/CRT might also affect the binding efficiency of DREBs. For example, Maruyama et al. [24] found that CBF3 bounds to A/GCCGACNT more efficiently than to A/GCCGACNA/G/C. Among the 302 inducible genes by low temperature in the wildtype Arabidopsis, 85 of them were induced by CBF2/DREB1C in transgenic Arabidopsis under normal growth temperature [25]. The *rd29A* promoter has five conserved DRE cis-elements [26]. In this study, the conversion of ACCGAC to GCCGAC of the third upstream core DRE cis-element of *rd29A* promoter nearly abolished the transcriptional activation of PvDREB1C. Furthermore, among the four up-regulated downstream functional genes in *PvDREB1C* transgenic plants under salt stress, three of their −2 Kb promoter regions (*NtSOD*, *NtPOD*, and *NtERD10B*) contain at least one ACCGAC sequences (see Additional file 1). Together, this result suggested that PvDREB1C preferentially activated those promoters with the core DRE cis-element of ACCGAC. Such information shall be useful for understanding the full picture of its downstream target gene networks using ChIP-seq and RNA-seq approaches in the future.

## Conclusions

It is inevitable to grow switchgrass in barren, saline and other types of marginal land for the purposes of biofuel and forage feedstock crop production and water and soil conservation. The current results showed that *PvDREB1C* played opposite roles in cold and salt tolerance, and supported that *PvDREB1C* could be used for improving switchgrass salt tolerance, but particular attentions shall be paid to its negative role in cold tolerance.

## Methods

### Plant materials

Switchgrass line ‘HR8’ [27] selected from the ecotype ‘Alamo’ was used to study the *PvDREB1*s expression levels. The plant growth condition was the same as previously reported with a 14 h photoperiod and the growth temperature set at 30/20 ± 3 °C (day/night) [28].

The tobacco (*Nicotiana tabacum* and *N. benthamiana*) plants were grown in pots with peat soil, and were maintained in growth chambers set at 23/20 °C (day/night) with a 12 h photoperiod and photoactive radiation of 350 μmol photons m^−2^∙s^−1^ for 4 weeks.

### Gene cloning and vector construction

Putative cold-inducible *DREB1* (*PvDREB1s*) genes were pooled out from the switchgrass genome database (*Panicum virgatum* v1.1, DOE-JGI) that were homologous to four rice cold-responsive OsDREB1 genes. These PvDREB1s were named as DREB1A-1 (Pavir.J03891.1), DREB1A-2 (Pavir.Ba01280.1), DREB1B-1 (Pavir.J19655.1), DREB1B-2 (Pavir.Ba01283.1), DREB1C (Pavir.J35991.1), DREB1G-1 (Pavir.Ab02685.1), DREB1G-2 (Pavir.Aa00912.1) and were amplified by PCR using the Q5 PCR mix (New England Biolabs, Beverly, MA) with the annealing temperature set at 61 °C for 30 amplification cycles. The gene was cloned into an entry vector and then to the plant expression vector pEarlyGate103 [29].

The pCAMBIA1302-Pro:GUS-polyA vector was constructed for promoter analysis. In brief, the GUS-polyA fragment was amplified by PCR from the pCAMBIA1305.1 vector with the flanking PstI & HindIII sites, cloned into pENTR/D vector, and sequenced. Then the fragment was inserted into the corresponding restriction enzyme sites of pCAMBIA1302. The resultant pCambia1302-Pro:GUS-polyA vector has a number of restriction enzyme sites (EcoRI – SacI – KpnI – XbaI – SalI - PstI) upstream of the GUS (*UidA*) gene for the insertion of target promoter.

The *rd29A* (AT5G52310) promoter was cloned from Arabidopsis ecotype ‘Columbia’, and sequenced. Mutations were introduced into the DRE/CRT cis-element to result in A/GCCGAC (sequences as shown in data S1). These five promoters were sub-cloned into the EcoRI & KpnI sites of pCAMBIA1302-Pro:GUS-polyA.

### Switchgrass stress treatment and qRT-PCR analysis

The switchgrass plantlets at the height of ~30 cm were used for the following treatments. In brief, three fully expanded leaves (the 3rd leaves from the top) from different plantlets were pooled as one sample, and the sampling time were set at 0, 0.5, 1, 2, 4, 8, 12 and 24 h after cold (4 °C), 20% PEG, or 250 mM NaCl treatment, respectively.

The total RNA was isolated from switchgrass leaves using RNApure fast isolation Kit (YuanPingHao, Tianjin, China). The first strand cDNA was synthesized with 1 μg RNA using the PrimeScript^RT^ Reagent Kit after the removal of gDNA (Takara, Dalian, China). The qRT-PCR was performed on a Roche LightCycler480 II machine (Roche Diagnostic, Rotkreuz, Switzerland) using the SYBR Green I Master Reaction System (Roche Diagnostic). The PCR condition was set as follows: 10 min at 95 °C for initial denaturation, and 40 cycles of PCR (95 °C for 15 s, 58 °C for 15 s, and 72 °C for 20 s). Three biological replicates and two technical replicates were performed. Relative expression levels were calculated using the 2^-ΔΔCT^ method with *PvFTSH4* as a reference gene [30]. The qRT-PCR primers used in this study were shown in Table 1.

**Table 1:** Primers used in this study

Primer sets	NCBI Accession No. for the target gene	Primer Sequence (5′-3′))	Purpose
*PvDREB1C_CDS*	Pavir.J35991.1	ATACGGATCATGGAGTACGACGAGCAGGAG	Cloning of *PvDREB1C*
		TGATAAGCTTGTCGTAGTAGCTCCACAGCGTC	
*35S_F1 & GFP_R1*	–	TCCACTGACGTAAGGGATGAC	PCR verification of transgenic tobacco
		AGAATTGGGACAACTCCAGTG	
*PvDREB1C_qRT*	Pavir.J35991.1	GGAGTACGACGAGCAGGAGT	qRT-PCR
		GGTCTCCCGGAACTTGGT	
*NtSOD*	AB093097	AGCTACATGACGCCATTTCC	qRT-PCR
		CCCTGTAAAGCAGCACCTTC	
*NtPOD*	D11396.1	GCTGTTCGACGAGTTGTTAACAG	qRT-PCR
		CTCTGGCTGAGTTGTTGTTGG	
*NtAPX1*	AU15933	CAAATGTAAGAGGAAACTCAGAGGA	qRT-PCR
		CAGCCTTGAGCCTCATGGTACCG	
*NtcAPX*	D85912	CTGGAGTTGTTGCTGTTG	qRT-PCR
		GGTGGCTCTGTCTTGTC	
*NtNHX4*	XM_016617731	CAAGAACTTCCGCACCCAC	qRT-PCR
		GCAGTATCAAACGCAGAGGACC	
*NtSOS1*	XM_016611194	CAAATGTTATCCCCCGAAAGC	qRT-PCR
		CGGAGAACCTGAGGAAATGTGA	
*NtERD10B*	AB049336	CAATTTAGTGCAGGCCAGGC	qRT-PCR
		GGTCCATGGTGGCCAGGAAG	
*NtPAO*	AB200262	GCTGCTCTGTCGTCATA	qRT-PCR
		TATCCTGCCACCTATCTTATC	
*NtCuAO1*	DQ873385	TATGGCAGTTGATTGTAAGC	qRT-PCR
		TGACGAGCAGAGAATGG	
*NtCuAO2*	AB289457	TATGGCAGTTGATTGTAAGC	qRT-PCR
		CCAATGACGAGCAGATAAAGT	
*NtUbiquitin*	XM_016580871	TCCAGGACAAGGAGGGTAT	qRT-PCR
		CATCAACAACAGGCAACCTAG	

### Tobacco genetic transformation and transient assay

All binary vectors used in this study were electro-transformed into the *Agrobacterium tumefaciens* strain ‘AGL1’. The genetic transformation of tobacco was conducted as previously described [31]. The transgenic plants were screened by 15 mg∙L^−1^ bialaphos and the regenerated plants, once rooted in soil, were verified by PCR.

The relative binding efficiency of PvDREB1C to the above described five *rd29A* promotors was tested using a transient assay method on mature leaves of 4-week-old *N. benthamina* plants. In brief, pEarleyGate103-*PvDREB1c*/AGL1 and pCAMBIA1302-*RD29A_*Pro: GUS-polyA/AGL1 were mixed together, and co-injected into tobacco leaves. The amount of injected solution was measured using a labelled syringe and was kept the same for all treatments. The empty vector, pCAMBIA1302-Pro:GUS-polyA without any promoter upstream of the *UidA* (*GUS*) gene, was used as the negative control. Leaves injected with the infection solution (1/2 MS, 3% sucrose and 100 μM acetosyringone, pH 5.7) were used as background controls. Each testing point was measured with 15 biological replicates. After 24 h, the injected leaves were sampled and measured for GUS activity using a method as described by Fior et al. [32].

### Evaluation of transgenic plants exposed to low temperature and salt stress

One and a half month old T2 transgenic and wildtype tobaccos were grown in pots and used for cold and freezing tolerance analysis: (1) for 4 °C cold tolerance, 18 plants of each line were moved into one growth chamber with temperature set at 4 °C and under light of 200 μmol photon m^−2^ s^−1^ for 24 h; (2) for freezing tolerance, another batch of tobacco plants (18 plants of each line) were moved to the growth chamber with temperature gradually decreased to −4 °C (lowering two degrees per hour starting from 4 °C), and maintained at −4 °C for 3 h. The plants were then moved back to optimum growth condition and their survival rates were counted after recovery for 2 d [33].

T2 transgenic plants were used for the salt tolerance. One and a half month old tobacco plants grown in pots were also used for salt tolerance assessment by watering with 200 mM NaCl for 25 days. Plants normally watered were used as controls.

### Measurement of physiological parameters

#### Electrolyte leakage

The leaf electrolyte leakage (EL) was measured according to Jiang and Huang [34]. In brief, leaves at the same positions were detached and incubated in 30 ml distilled deionized water. The initial level of EL (C_i_) was measured using a conductance meter (Thermo Scientific, Baverly, USA) after shaken for 24 h at room temperature. The leaf tissue was then killed in an autoclave at 121 °C for 30 min, and incubated for 24 h on a shaker for measuring the maximum conductance (C_max_) of the incubation solution. Relative EL was calculated as EL = (C_i_/C_max_) × 100.

#### Osmotic potential

Osmatic potential was measured using an osmatic potential determinator Vapro Model 5520 (Wescor, Logan, Utah). The leaf tissue was soaked in distilled water for 6 h, then in liquid nitrogen for 1 min, and on ice for another 30 min. Finally, the leaves were ground into juice for measurement.

#### H_2_O_2_ content

The H_2_O_2_ content was measured according to Sergiev [35]. In brief, seedlings were extracted with 2 ml cold 5% (*w*/*v*) TCA. The extraction was then centrifuged at 12,054 g for 15 min. The 0.5 ml supernatant was mixed with 0.5 ml PBS (0.1 M, pH 7.0) and 1 ml KI (1 M), and then incubated at room temperature in the dark for 1 h. The absorbance of the mixture was measured at 390 nm and the concentration of H_2_O_2_ was calculated against the standard curve.

#### SOD, POD and APX activities

A total of 0.2 g leaves were ground in 4 ml extracting solution (3 ml 50 mM PH7.8 PBS and 1 ml 1 mM EDTA·2Na) and the homogenate was centrifuged at 15,000 g for 30 min at 4 °C, the supernatant was collected for enzyme activity analysis. The activities of SOD, POD and APX were measured by spectrophotometry according to the method described by Giannopolitis et al. [36], Zheng et al. [37] and Hossain et al. [38], respectively.

### Na^+^, K^+^ and Ca^2+^ contents

The leaves and roots of transgenic plants and wildtype treated with or without 200 mM NaCl were used for the Na^+^, K^+^ and Ca^2+^ content measurement according to Shukla et al. [39].

### Statistical analysis

Data in this study were statistically analyzed using one-way ANOVA, and the means were compared by Duncan test at a significance level of 0.05 by using SPSS (version 12, SPSSInc., Chicago, IL, USA). Data were shown as mean ± SE. Different letters above the bar in figures represented statistically significant difference at the level of 0.05.

## Additional file

Additional file 1: The mutated sequences of *rd29A* promoter used in this study. (XLSX 26 kb)

## Abbreviations

APX: Ascorbate peroxidase
CBFs: C-repeat-binding factors
DRE: Dehydration-responsive element
DREB: Dehydration-responsive element binding factor
EDTA: Ethylenediaminetetraacetic acid
EL: Electrolyte leakage
PBS: Phosphate buffer saline
POD: Peroxidase
SOD: Superoxide dismutase
TCA: Trichloroacetate
